# Volume discrimination of nanoparticles via electrical trapping using nanopores

**DOI:** 10.1186/s12951-019-0471-5

**Published:** 2019-03-14

**Authors:** Akihide Arima, Makusu Tsutsui, Masateru Taniguchi

**Affiliations:** The Institute of Scientific and Industrial Research, Osaka University, 8-1 Mihogaoka, Ibaraki, Osaka 567-0047 Japan

**Keywords:** Nanopore, Electrophoresis, Biosensor, Resistive pulse measurement

## Abstract

Electrophoretic capture of an oversized object on a solid-state nanopore is a useful approach for single-particle analyses via post electrical and optical measurements. Here we report on nanoparticle discriminations by the volume through combining this nanopore trap method with the cross-membrane ionic current measurements. We investigated ion transport through a pore channel being partially occluded by an electrophoretically-drawn nanoparticle at the orifice. We found distinct difference in the amount of current blockage by particles of different sizes. Multiphysics simulations revealed dominant contribution of particle volume over the other properties. We also demonstrated single-particle discriminations of two different sizes in a mixture solution. The present results demonstrate that this electrical capturing is a promising technique to immobilize a target at a single particle level that concomitantly offer wealth of information concerning their volume.

**Electronic supplementary material:**

The online version of this article (10.1186/s12951-019-0471-5) contains supplementary material, which is available to authorized users.

## Introduction

Nanopore analyses are a simple and strong method for a particle characterization that enables evaluations of various parameters such as the shape, volume, and surface charge density [1–8]. In the measurement, electrophoretic entering of analytes into the nanoscale conduit causes a short-time decreasing of the cross-pore ionic current, and the associated blockade current is used for studying the physical characteristics of individual analytes [9–12]. This method, however, cannot be used for repetitive measurements of single-particles unless additional probes are incorporated to regulate the fast translocation motions such as dielectrophoresis [13], optical tweezer [14], and a magnetic force [15].

**Fig. 1:**
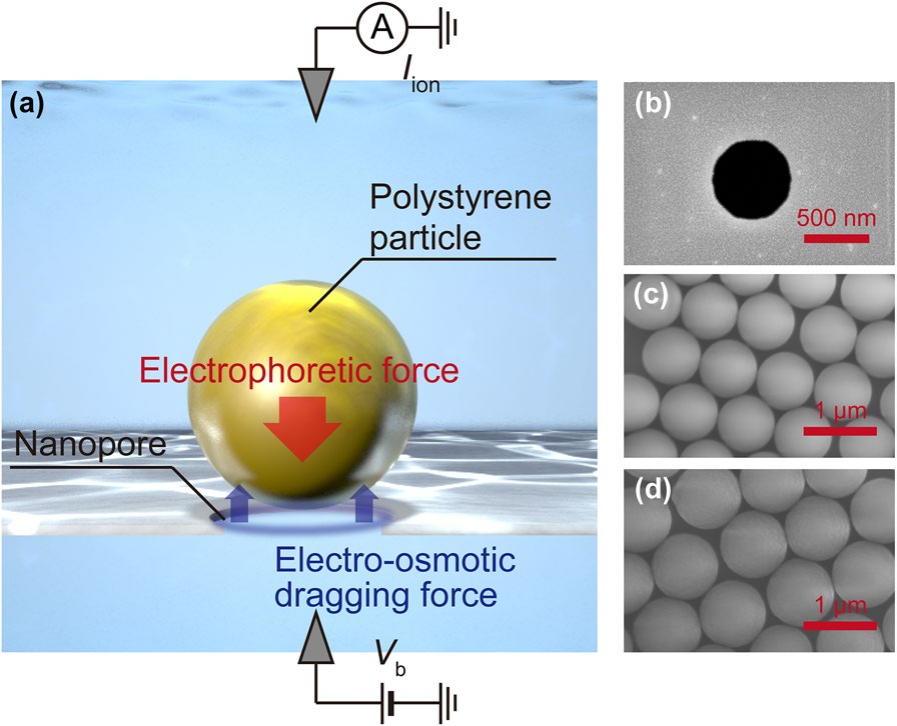
**a** Schematic image of nanopore trapping method and **b**–**d** scanning microscopic images of pore and particles. **b** A 600 nm-sized nanopore was employed for capturing carboxylated-polystyrene particles sized **c** 780 nm and **d** 900 nm

On the other hand, nanopore trap method is a more simple and facile strategy for the target immobilization at a single-particle level (Fig. 1a) [16, 17]. This method utilizes a nanopore with a diameter smaller than that of analytes of interest. Unlike conventional resistive pulse measurements [18], the targets are not able to pass through the pore but become immobilized at the orifice under the applied electrophoretic voltage. Previous works [16] have proven the ability of discrimination between surface charges of equi-sized nanoparticles using a low thickness-to-diameter aspect ratio nanopore. Here, in this report, we investigated the feasibility of the nanopore trap method for discriminating particles by the volume. We repetitively measured the ionic current blockage of two nanoparticles having different sizes. As a result, larger particles were found to block the ion transport more effectively whereby enabled discriminations of single-particles by the volume. This finding can open the prospect for tracing target condition to gain wealthy information about the trapped analyte such as in the situation of cell growth and shows the advantage in the incorporation of additional probes such as tunneling current via nanoelectrode employing capability of the method as a delivery and capture system [19–22].

## Methods

The fabrication process of a nanopore is described elsewhere [8, 17]. Briefly, 20 mm × 20 mm sized silicon chips constructed with three layers, SiN/Si/SiN = 50 nm/0.5 mm/50 nm, were used as substrates. Through a reactive ion etching (RIE) for removing partial area of SiN layer on one side of the surfaces and following anisotropic wet etching of Si in KOH aq., a 50 nm-thick SiN membrane was prepared. After forming metal patterns (thickness: Cr/Au = 2 nm/30 nm) by photolithography, radio-frequency magnetron sputtering, and lift off in *N*, *N*-dimethylformamide, a 600 nm-sized pore was excavated using electron-beam lithography and RIE (Fig. 1b) in the thin membrane through using the metal patterns as markers.

For the nanopore measurements, polydimethylsiloxane (PDMS) blocks having microchannels were attached on both sides of the chip. Suspension of target particles was then injected through inlet and outlet holes penetrated in the polymeric blocks. After the injection, Ag/AgCl electrodes were set on the both blocks for application of the electrophoretic voltage *V*_b_ and measuring the ionic current *I*_ion_ using Keithley 6487 picoammeter/source (Tektronix, Inc.) under the particle trap control by the handmade program using Visual Basic 6.0.

As target analyte, two carboxylated-polystyrene particles (PS-COOH) with diameter 780 nm and 900 nm (Fig. 1c, d, Thermo Fisher Scientific, Inc.) are utilized after dispersion into TE buffer (10 mM Tris–HCl, 1 mM EDTA). Their ζ-potentials were measured using Zetasizer Nano ZS (Malvern Panalytical Ltd., Zetasizer software ver. 7.12). For each particle, we obtained the values of − 73.8 mV and − 68.6 mV, respectively. Note that these oversized particles are not capable of translocating through a 600 nm pore.

## Results and discussions

As shown in Fig. 1a, the principle of a nanopore trapping method is based on a physical blocking of ion transport through a pore channel. The presence or absence of the particles at the pore can be checked by monitoring temporal changes in *I*_ion_: when a particle is captured, the current rapidly decreases due to a partial blocking of ion transport through the pore. In trapping, a particle is floating at a vicinity of the channel as the result of balance between electrophoretic force and drag force of electroosmotic flow. Both of these forces are proportional to the amplitude of voltage. The resistance of nanopore system can be described as a sum of two elements; *R* = *R*_pore_ + 2*R*_acc_. In this equation, *R*_pore_ = 4*ρL*/π*d*_pore_^2^ and *R*_acc_ = *ρ*/2*d*_pore_ with electrical resistivity ρ, pore diameter *d*_pore_, and thickness *L* are pore resistance which means the component from inside of a pore and access resistance from electrodes to pore orifice, respectively [23–28]. In the measurement of *I*_ion_ using a low aspect ratio nanopore, the factor of *L*/*d*_pore_^2^ in *R*_pore_ would approach to zero and the total resistance could be approximated as *R*_acc_. Therefore, the amplitude of current blockades in trapping strongly concerns with the volume and the surface charge density of entering particles.

**Fig. 2:**
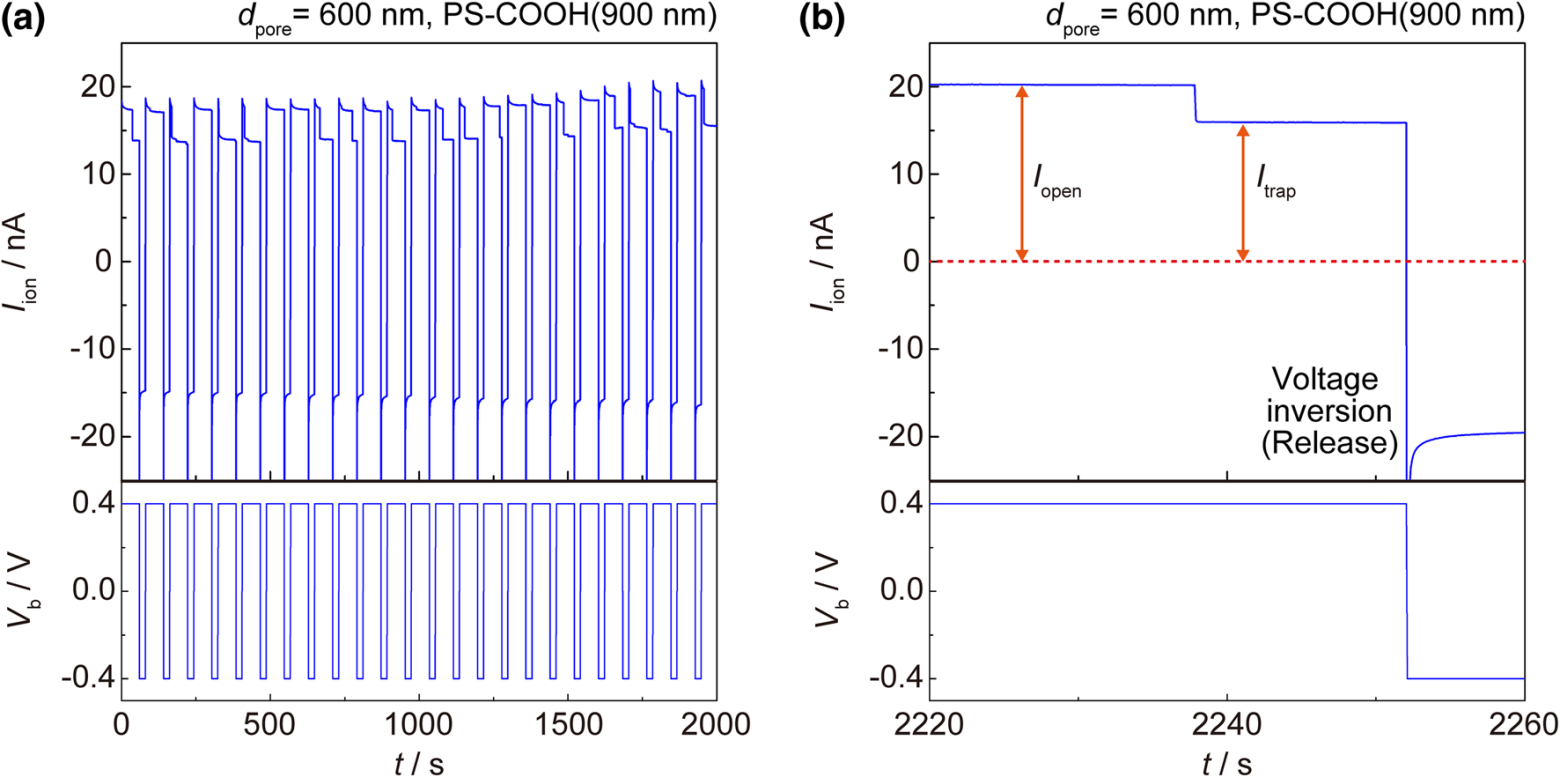
**a** Typical consecutive current traces of electrophoretic particle trap and **b** magnified view of a single trace showing open pore current *I*_open_ and suppressed *I*_trap_. Trapping/detrapping processes are controlled by inversion of *V*_b_

Typical ionic current traces during a repetitive nanopore trapping is shown in Fig. 2a and focused in Fig. 2b. Before trapping, the pore is fully open and the current values represent a constant ionic current *I*_open_. When a particle is captured, the flow of ions is suppressed and the drop to *I*_trap_ is observed [16, 17, 29]. As complete sealing should lead *I*_ion_ to zero, the non-zero *I*_trap_ indicates a particle floating at the vicinity of the pore because of the drag force of electroosmotic back flow antagonizing to the electrophoretic forces of the negatively-charged particle. After trapping, the particle can be released by a simple inversion of *V*_b_. The consecutive traces indicate long-term stability and reproducibility of the trapping/detrapping processes.

**Fig. 3:**
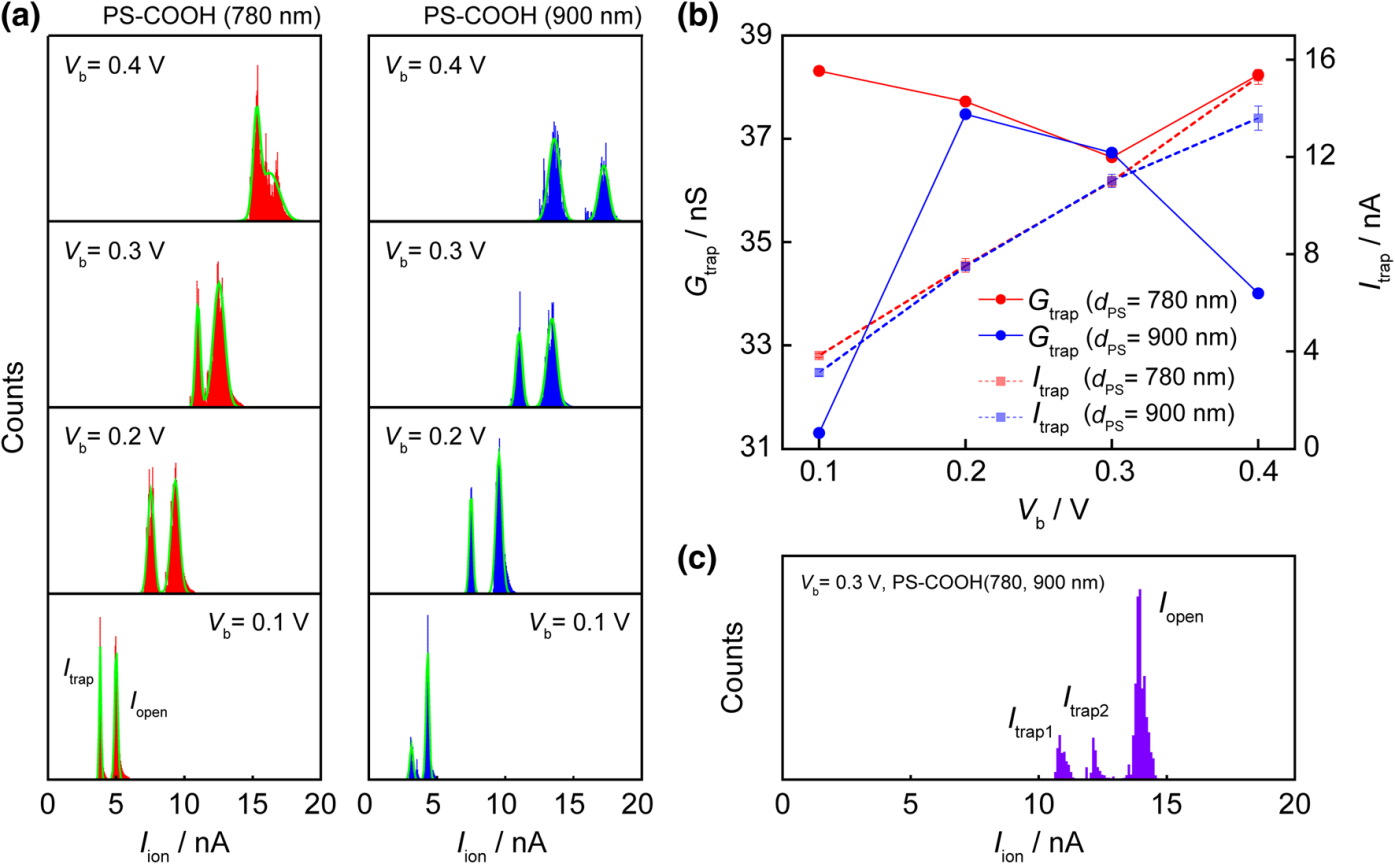
Voltage dependence of **a** current histogram, **b** occlusion amplitude of single-sized particles. **c** Current distribution of multiple particles trapping

Figure 3a shows voltage-dependence of *I*_ion_ in the course of trapping processes for the both particles. At every *V*_b_ conditions tested, bimodal distributions corresponding to *I*_open_ and *I*_trap_ were obtained. To evaluate the dependence of ionic current suppressions on the voltage conditions, the conductance *G*_trap_ = *I*_trap_/*V*_b_ was derived for the peak value in Fig. 3a. The voltage-dependency of *G*_trap_ for each particle is shown Fig. 3b. Despite of the similarity at *V*_b_ = 0.2 and 0.3 V. The displacement is observed at *V*_b_ = 0.1 and 0.4 V indicating further suppression by the particles sized 900 nm. Assuming that linear relationship that higher (lower) voltage provide stronger (weaker) electrophoretical withdrawal of particles, this trend is counterintuitive. This diremption could be explained by contribution of electroosmotic flow. In trapping, the particles is at the vicinity of a pore orifice sufficiently under electrophoretic force. However, at the same time, it enables effective application of dragging force of electroosmosis. This exception suggests a shift of the equilibrium between the two counteractive forces. Consequently, values of *I*_trap_ has close relationship with the position of the trapped particle. In the quantitative approach of our previous work [16], the order of these forces are nearly equal and electrophoretic force is little larger. Hence, electroosmotic flow affect the particle position certainly. However, nanopore trapping system based on the force-balance is too complicated and further studies are needed in order to shed light on the detailed estimations. Besides, we also adopted a nanopore trapping method to mixture of these particles (Fig. 3c). From current traces, a trimodal histogram was obtained. Each peak can be ascribable to *I*_open_, *I*_trap1_ by the 780 nm-sized particle, and *I*_trap2_ that of 900 nm-sized one. For assignment, *I*_trap_ was utilized from each value at *V*_b_ = 0.3 V. As a result, the two values, 10.89 ± 0.20 and 12.17 ± 0.09 nA, were acquired as *I*_trap1_ and *I*_trap2_, respectively. Compared with the *I*_trap_ values of the individual particle measurements in Fig. 3b, *I*_trap(780 nm_) = 10.99 ± 0.21 nA and *I*_trap(900 nm_) = 11.02 ± 0.27 nA and its potential of particles discrimination is confirmed. The results prove the possibility of discrimination of captured objects from their volume and surface charge properties.

**Fig. 4:**
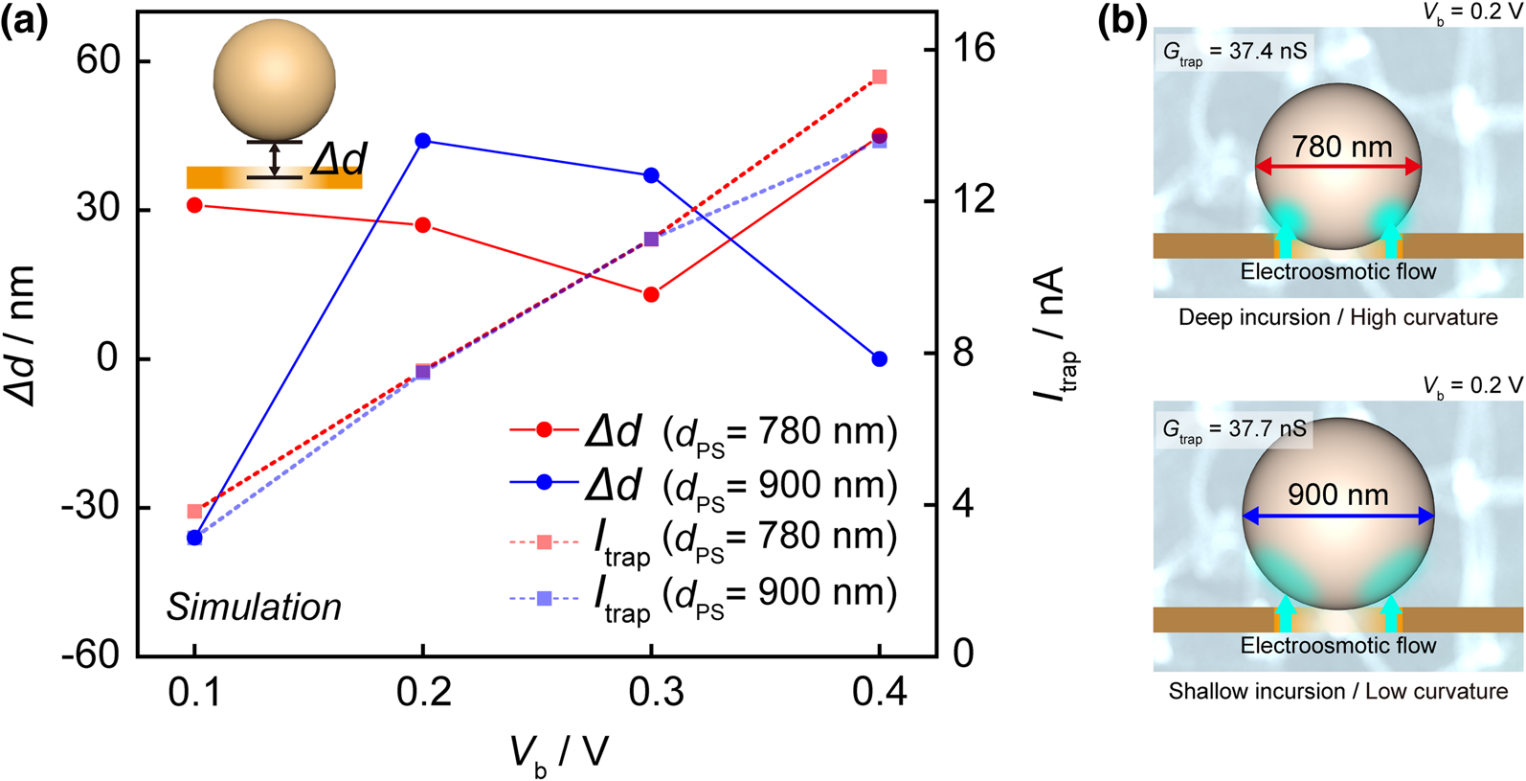
**a** Particle positions and ionic currents in trapping derived from multiphysics simulations. **b** The commensuration between contribution of particle curvatures and positions at *V*_b_ = 0.2 and 0.3 V

To shed light on the relationship between *I*_trap_ and positions of a particle, multiphysics simulation was conducted (Fig. 4 and see Additional file 1) [16, 17]. We approximate the pore-particle distance (*Δd*) by comparisons between the experimental *I*_trap_ with simulated ones. Interestingly, we found that under different *V*_b_, the trajectories of particles are quite different. Specifically, in both cases, it seems that *Δd* tends to decrease with the voltage. It is inspiring that the shapes of voltage dependency of *Δd* is similar with that of *G*_trap_. However, in the same manner as *G*_trap_, the displacement also appears in *Δd* at the *V*_b_ = 0.2 and 0.3 V. In other words, these particles show similar ionic suppression despite of deeper incursion of the particle sized 780 nm. It seems that 900 nm-sized particle cannot invade into a pore as 780 nm due to stronger application of electroosmotic flow, whereas lower curvature occupies transport pass for ions more effectively. It demonstrates ionic transports inhibited equally as a result of commensuration between contribution of particle curvatures and positions.

For further evaluation of relationship between the amplitude of ionic current suppression and blocking factor; volume and surface charge density, we applied nanopore trapping method to various particles (*d*_PS_ = 0.78, 0.90 μm (Thermofisher Scientific Inc.) and 0.79, 0.99 μm (Polyscience, Inc.). If the surface charge density is the dominant factor of ionic current suppression, 0.99 μm PS-COOH (ζ-potential: − 81.9 mV) should show the smallest *I*_trap_. However, the comparison showing in Additional file 1: Figure S2 reveals smaller particle which has relatively weak charge (*d*_PS_ = 0.78 and 0.79 μm) can block ionic flow more effectively. This means that the certain superior of volume in ionic blockade. In addition, we employed a smaller pore sized 300 nm to trap two particles of *d*_PS_ = 0.49 and 0.52 μm (ζ-potentials: − 61.4 and − 62.7 mV, respectively) in 0.4× PBS buffer (Additional file 1: Figure S3a). Despite the similarity of their ζ-potentials, the smaller particle demonstrates larger suppression. This result proves our assumption; the dominant cause of target volume on trapping current. Furthermore, we also attempted to capture the large particles (*d*_PS_ = 0.78, 0.79, 0.90, and 0.99 μm) using this smaller pore (Additional file 1: Figure S3b). In spite of the larger ζ-potentials than two particles trapped steady by this pore, the ionic currents in capturing shows great fluctuations indicating an incomplete immobilization since the effective electrical field is smaller and the contribution of electroosmotic force become large relatively. It reveals that the limitation of nanopore trapping is depend on the size of analyte.

## Conclusion

In conclusion, it was revealed that a nanopore trapping method has ability of volume-specific discrimination with similarity of surface charge. In the particle trapping process using a pore, the factor determining ionic suppression is mixing of surface charge and particle size and their priority would appear in the similarity of another. The amplitude of ionic flow is reflected a particle properties of analytes representing a volume and the usefulness as status diagnostic method for single-particle is demonstrated. Besides, contradistinction between similarity of ionic blockade and dissimilarity of trajectory indicated detail electrokinetic factor in nanoscale. This result also suggests the possibility to serve as position modulator of micro-nano scale by a simple control of applied voltage in liquid condition and provide extensibility of sensing in such conditions.

## Additional file

**Additional file 1.** Multiphysics simulation of particle blocking events, Distribution of ionic current in trappings of various particles, and Particle trapping employing a smaller nanopore.
