# Supplementary material for: Volume discrimination of nanoparticles via electrical trapping using nanopores
Source: J Nanobiotechnology. 2019 Mar 14;17:40. doi: 10.1186/s12951-019-0471-5 (PMC6419447; doi:10.1186/s12951-019-0471-5)
Supplement: Supplementary file 1 — Additional file 1. Multiphysics simulation of particle blocking events, Distribution of ionic current in trappings of various particles, and Particle trapping employing a smaller nanopore. [file 12951_2019_471_MOESM1_ESM.pdf]

Additional file for

Volume discrimination of nanoparticles via electrical trapping using nanopores

[*1] Akihide Arima, [*1] Makusu Tsutsui, [1] Masateru Taniguchi

[1] The Institute of Scientific and Industrial Research, Osaka University, 8-1 Mihogaoka, Ibaraki, Osaka 567-0047, JAPAN

[*]Authors to whom correspondence should be addressed. Electronic address: arima@sanken.osaka-u.ac.jp, tsutsui@sanken.osaka-u.ac.jp

The additional file includes:

1. **Multiphysics simulation of particle blocking events**
2. **Distribution of ionic current in trappings of various particles**
3. **Particle trapping employing a smaller nanopore**

Multiphysics simulation of particle blocking events

For estimation of an open pore and blocking current by a polystyrene particle, we performed a multiphysics simulation through a model described in the following: Navier-Stokes equation for liquid flow through a nanopore, Poisson equation for the electrical potential distribution, and Nernst-Plank equation for ion transport in the nanopore system. Two-dimensional axisymmetrical multiphysical model of a nanopore axial-radial (z-r) plane was built in COMSOL® for solving the above equations (Figure S1) [S1]. In our nanopore system, which has low-thickness-to-diameter aspect ratio, the access resistance of the chamber become much larger than a pore resistance [S2]. Therefore, our simulation model was constructed with r_{up}= 3000 nm and r_{down}= 500 nm to calculate the access resistance correctly. Open boundary conditions were used at the ends of *cis* and *trans* chambers for Navier-Stokes equation. Bulk ion concentration of $C_{Tris\text{-}Cl}$ = 7.13 mM of TE buffer was assumed at those two ends. Electrically, the end of *cis* chamber was kept at 0 V, and *trans* chamber at V_b. The ion mobilities of μ_{Cl} = $7.911\times10^{-8} m^2/s\cdot V$ and μ_{Tris} = $1.980\times10^{-8} m^2/s\cdot V$ were used [S1]. The positions of a PS particle are derived from the comparisons between experimental blocking current I_{block} and simulated value with the sequential change of a particle position.

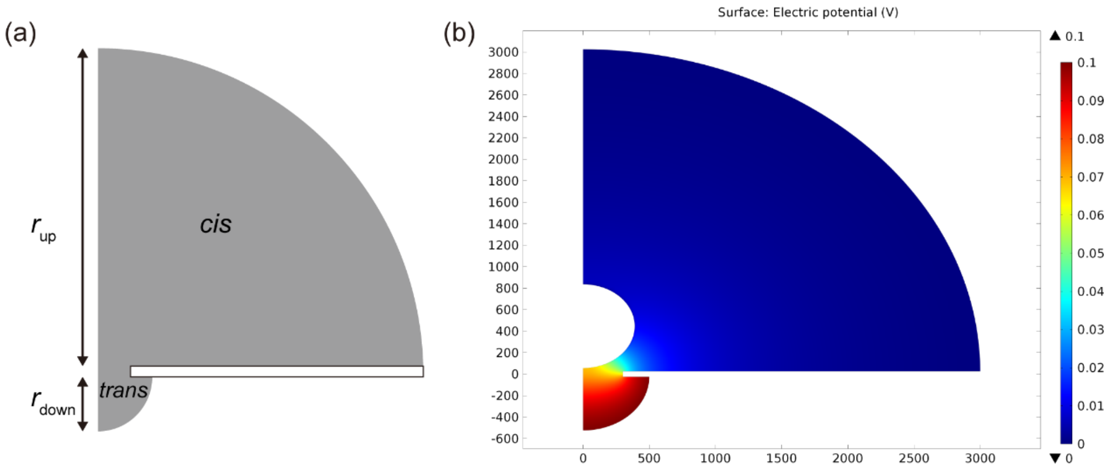

Figure S1. The assessment of particle positions in nanopore trapping. (a) The model of nanopore system for COMSOL®. (b) Potential map at V_b = 0.1 V in the simulation of a particle trapping (PS-COOH (780 nm) with Δd = 31 nm)

Distribution of ionic current in trappings of various particles

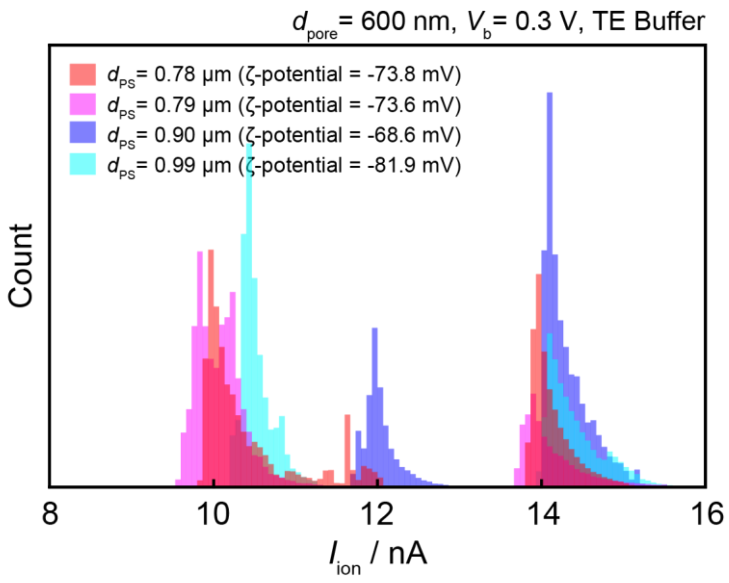

Figure S2. Ionic current histograms in trapping/detrapping processes of various particles.

Particle trappings employing a smaller nanopore

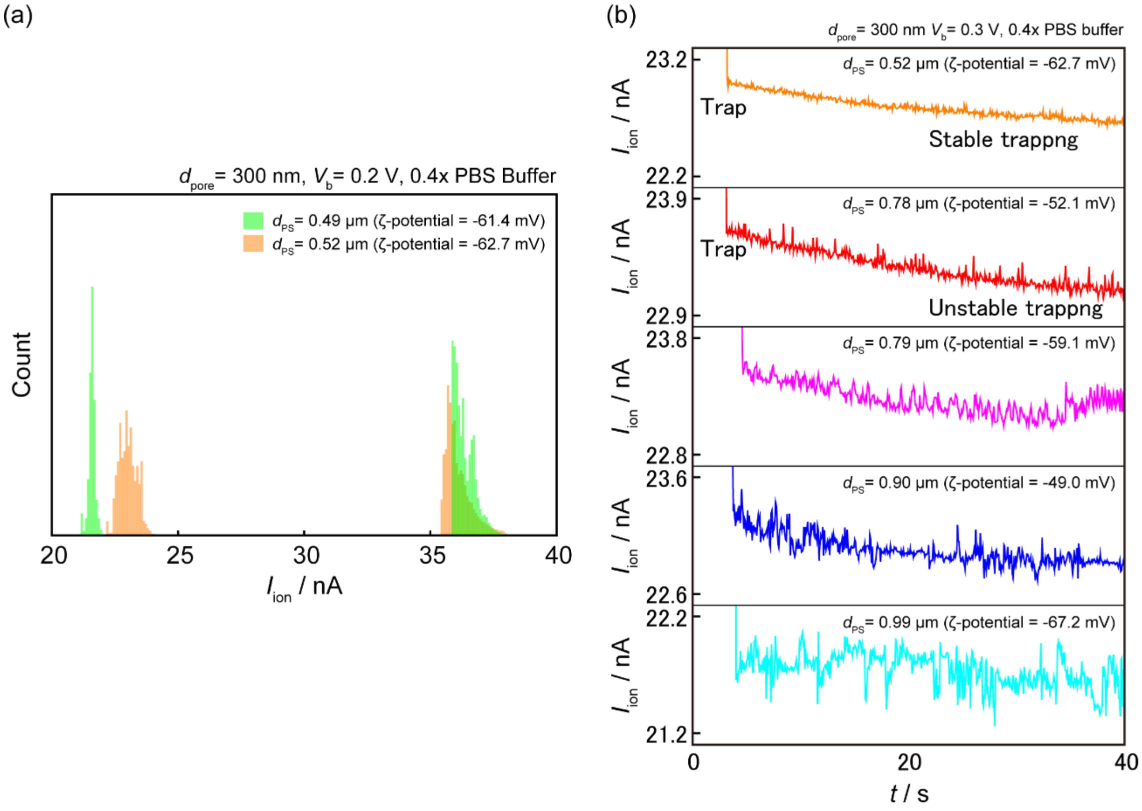

Figure S3. (a) The distribution of ionic current in electrical captures of particles which have different diameter and similar ζ-potential using a 300 nm-sized nanopore. (b) Typical current traces in trapping concerning various particles indicating capture limit. Only 0.52 μm polystyrene beads was captured stably.

Additional references

[S1] Tsutsui M, Maeda Y, He Y, Hongo S, Ryuzaki S, Kawano S, Kawai T, Taniguchi M. Trapping and identifying single-nanoparticles using a low-aspect-ratio nanopore. *Appl Phys Lett.* 2013;103:013108.

[S2] Arima A, Tsutsui M, He Y, Ryuzaki S, Taniguchi M. Electrical trapping mechanism of single-microparticles in a pore sensor. *AIP Advances*. 2016;6:115004.
